# Transidentification in Adolescents and Young Adults: Understanding Parental Concerns to Improve Psychological Support for Families

**DOI:** 10.1007/s10508-025-03139-w

**Published:** 2025-08-12

**Authors:** Anna Cognet-Kayem, Alexandre Ledrait, Capucine Lamoureux, Olga Megalakaki, Céline Masson

**Affiliations:** 1 Department of Psychotherapy, Sigmund Freud University, 75015 Paris, France; 2 Laboratoire de Psychologie Caen Normandie (LPCN – EA7452), University of Caen Normandie, Caen, France; 3 Paris, France; 4 Psychology Department, CRP-CPO (UR 7273), Université Picardie de Jules Verne, Amiens, France; 5 Psychology Department, CHSSC (UR 4289), Université de Picardie Jules Verne, Amiens, France

**Keywords:** Transidentity, Puberty, Parenthood, Transgender, Gender dysphoria

## Abstract

This study explores the experiences and perceptions of parents of adolescents and young adults who identify as transgender, particularly focusing on the anxiety and ambivalence that some parents experience regarding their child’s disclosure of a transgender identity. While existing research on transidentity among minors has addressed challenges within family dynamics, it has not deeply examined the underlying reasons for parental hesitation in supporting their child’s transition. To fill this gap, we collected and analyzed letters from 60 parents using the Interpretative Phenomenological Analysis method. The findings reveal that parents often struggle with profound ambivalence, balancing fears about the potential consequences of their child’s transition with a desire to provide support. The disclosure of a transgender identity frequently challenges parent–child communication, resulting in perceived disconnects and concerns about the child’s mental health. Parents actively sought guidance from healthcare professionals but often felt disappointed by consultations they perceived as lacking thoroughness. Based on these findings, we recommend adopting an exploratory and psychotherapeutic approach in healthcare, fostering individualized and supportive care for both families and transgender youth, in contrast to standardized, protocol-driven consultations that may overlook the complexity of family dynamics and psychological factors.

## Introduction

The sociopolitical context surrounding transidentity among young people can influence how both individuals and their families navigate questions related to gender identity and transition. While some environments promote acceptance and affirmation, others may discourage reflective discussions about an individual’s desire to pursue a transition, which often involves significant, life-altering decisions. In this context, parents who may seek to understand and support their child thoughtfully can sometimes feel stigmatized if their approach includes critical reflection or questions.

To address this complexity, it is essential to distinguish between gender dysphoria and transidentification. Gender dysphoria refers to the psychological distress arising from a mismatch between an individual’s experienced gender and their assigned sex at birth. This distress, if persistent, can lead to a desire for gender transition (Bouman et al., 2016). In contrast, transidentification draws from psychoanalytic concepts of identification, describing processes through which individuals internalize characteristics of others, shaping their sense of self (Ferenczi, 1933; Freud, 1937; Laplanche & Pontalis, 1973; Winnicott, 1958). This concept provides a framework for understanding how identity can be influenced by both internal dynamics and external factors, offering a holistic perspective on gender-related experiences.

Many parents seek a comprehensive understanding of their child’s experiences, especially when the disclosure of a transgender identity emerges without prior indicators of gender-related distress. This study aims to explore parental perceptions and experiences related to their child’s disclosure of transidentity, with the goal of enhancing psychological support strategies for families.

### Background of the Debate and Psychopathological Data

In recent years, there has been a notable increase in requests for medical transition among young people in Western countries, with some studies reporting significant growth rates (Barrett, 2015; de Graaf et al., 2018; Zucker, 2017). A particular trend has emerged: the increasing number of requests from adolescent girls, often described as occurring during adolescence without prior signs of gender-related distress. This phenomenon has been referred to as rapid onset gender dysphoria (ROGD) (Littman, 2018), contrasting with early onset gender dysphoria. While ROGD is not a formal diagnostic category, it has been used descriptively to explore patterns in the development of gender-related distress.

This shift raises questions about the contexts in which young people seek gender transition. Understanding these dynamics is complex, as the debate often contrasts two perspectives: the gender-affirmative approach and a more holistic approach to gender dysphoria. Proponents of the gender-affirmative approach suggest that mental health challenges in transgender youth are primarily linked to experiences of stigma, discrimination, and minority stress, advocating for early and supportive transition pathways as a means to improve well-being (Ghiasi et al., 2024; Reisner et al., 2016; Turban et al., 2021).

Conversely, advocates of a holistic approach emphasize the need to consider co-occurring neurodevelopmental and psychiatric conditions when supporting young people with gender dysphoria. They propose that gender-related distress may, in some cases, be intertwined with other psychological factors, such as autism spectrum disorder, attention deficit hyperactivity disorder, anxiety, eating disorders, depression, and trauma (Bailey & Diaz, 2025; Bechard et al., 2017; de Vries et al., 2010; Hall et al., 2021; Kozlowska et al., 2021; Kulatunga-Moruzi, 2023; Sapir et al., 2024; Sinai, 2022). From this perspective, comprehensive assessments are essential to understand the full range of factors contributing to an individual’s experiences.

While these approaches are sometimes presented in opposition, many clinicians integrate elements from both, seeking to provide individualized care tailored to the specific needs of each young person. Studies have highlighted both the potential benefits and risks associated with early medical transition, noting that outcomes can vary depending on individual circumstances (Cass, 2024; Schwartz, 2021; Withers, 2020).

Research also points to methodological limitations in some studies supporting early transition, such as small sample sizes, potential biases, and confounding variables (Clayton, 2023; Levine et al., 2022). This underscores the need for ongoing research to deepen our understanding of gender dysphoria and its complex interactions with mental health. A multidisciplinary approach, involving mental health professionals, young people, and their families, is essential to develop supportive strategies that promote well-being and psychological resilience (Masson et al., 2023).

To explore parental perspectives within this context, this study analyzed unsolicited, spontaneous letters from parents. Our research and our public alerts brought in parents who wanted to ask questions and, above all, bear witness to what was happening to their child. The aim is to better understand the nature of their perceptions and initial reservations regarding their child’s disclosure of transgender identity.

## Method

### Participants

The participants in this study were 60 parents of adolescents and young adults (AYAs) aged between 12 and 24 who identified as transgender. These parents voluntarily contacted the researchers at the Observatoire de la Petite Sirène (OPS), seeking to share their experiences and concerns.^1^ The testimonies were unsolicited, reflecting the spontaneous desire of these parents to express their perspectives.

A detailed overview of the demographic characteristics of the participants and their families is provided in Table 1.

**Table 1:** Overview of the demographic characteristics of the participants (*n* = 60)

Characteristic	Value
Total number of accounts	60
Average age of children	17
Median age of children	16
Sex distribution (female)	85% (*n* = 51)
Sex distribution (male)	15% (*n* = 9)
Parents in a couple	52% (*n* = 31)
Separated parents	23% (*n* = 14)
Widowhood	Rare
Missing family structure information	25% (*n* = 15)
Statements from mothers	73% (*n* = 44)
Statements from fathers	20% (*n* = 12)
Statements from both parents jointly	7% (n = 4)

### Measures and Procedure

The data for this study were collected from unsolicited, spontaneous written testimonies provided by parents. These testimonies were sent via email on a voluntary basis, without any invitation or solicitation from the researchers. This approach allowed parents to share their experiences and perspectives freely, without the guiding influence of pre-formulated questions.

In total, we received approximately 100 letters. The letters were received between 2019 and 2021, with an average year of submission being 2020. For the purposes of this study, we selected 60 testimonies that provided sufficient detail and coherence to form a consistent qualitative corpus. The length and depth of the responses varied, offering a range of personal narratives that reflected each family’s unique circumstances.

The parents who participated in this study voluntarily reached out to an association known for advocating a cautious approach toward the medicalization of minors. As a result, the testimonies may reflect a certain selection bias, predominantly representing narratives expressing concerns or hesitations rather than full acceptance. This context is important to consider when interpreting the findings, as it may influence the perspectives presented in the data.

### Ethical Considerations

Participants gave their informed consent by email, explicitly authorizing the use of their anonymized letters for research purposes. The email exchange included consent for the inclusion of their data in a scientific publication, with the assurance that all identifying information would be removed to guarantee confidentiality, in compliance with ethical standards for qualitative research. Identifiable information was removed before analysis to protect the privacy of both parents and their children. Since the data consisted of unsolicited, spontaneous testimonies sent voluntarily by parents, and no active recruitment or intervention was involved, the study did not fall under the jurisdiction requiring formal Institutional Review Board (IRB) approval under French regulations. According to the French Public Health Code, only research involving active participation of human subjects, such as biomedical acts or interventions, requires prior approval by an ethics committee (Comité de Protection des Personnes, CPP). As specified in Article R1121-1 of the Code de la santé publique (2023): “Research involving human subjects refers to research organized and carried out on human beings for the purpose of developing biological or medical knowledge.” Our study, based on voluntarily submitted testimonies and involving no recruitment, intervention, or experimentation, is thus considered to fall outside this legal framework—commonly referred to in France as being *hors loi Jardé*. In addition, the study complies with the CNIL’s Methodology of Reference MR-004 (2021), which governs the use of previously collected personal data in health research, provided anonymity, confidentiality, and participants’ rights are respected. However, the research adhered to ethical standards aligned with qualitative research practices, including strict confidentiality measures and the voluntary nature of participation.

### Data Analysis

The data were analyzed using Interpretative Phenomenological Analysis (IPA), a qualitative approach designed to explore how individuals make sense of their experiences. Given that this study focuses on parental perceptions and the meaning they attribute to their child’s disclosure of transgender identity, IPA was particularly suited to capturing the depth and complexity of these personal narratives.

The analysis involved a close reading of the testimonies to identify key themes and interpret the underlying cognitive and emotional processes shaping parental responses. Two researchers independently coded the data to enhance reliability, ensuring that emerging themes reflected both commonalities and individual variations in parental experiences. Discrepancies were resolved through discussion, and a third researcher reviewed the coding structure to ensure coherence and interpretative depth.

By focusing on the ways in which parents construct meaning around their child’s gender identity and transition, this methodological approach provides insights into the tensions, uncertainties, and evolving understandings that characterize these family dynamics. This interpretative lens allows us to move beyond a mere thematic description and instead explore how parents navigate conflicting emotions, societal discourses, and their own evolving beliefs.

## Results

### Main Categories

After reviewing the collection of accounts, we identified a common narrative structure that appears throughout the statements, generally following this sequence: the presentation of the family, the circumstances surrounding the disclosure of transgender identity (specific psychosocial functioning, symptomatology, life challenges), the surprise of relatives at the announcement, the parents’ attempts to understand (through discussions and information-seeking), disappointment related to specialist consultations, difficulties in maintaining a good relationship with their child, and requests for support from healthcare professionals.

The data were sorted into the following categories: sex of the adolescent or young adult (AYA), age of the AYA, parental situation, presence of siblings, which parent(s) provided testimony, presence of psychological follow-up prior to the disclosure, psychiatric diagnoses before the disclosure, triggers for the disclosure according to the parents, coexisting comorbidities alongside the disclosure, recourse to psychological consultations, recourse to specialized transgender consultations, number of consultations prior to diagnosis, social transition, experiences related to “passing,” use of puberty blockers, use of hormone therapy, surgical interventions, influence of social networks or peer groups, online activity, sexual orientation, and the psychological state of the AYA post-disclosure (see Table 1).

It is important to note that not all statements provided complete information for every category. The variability in the length and detail of each account reflects the diversity of family experiences and contexts. Consequently, some categories could not be consistently filled due to missing or unspecified information, highlighting the heterogeneity of the data collected.

### Thematic Analysis

The thematic analysis of the written statements highlights several essential elements of the parents’ experiences and the context in which their child’s transgender identity was disclosed. Several recurring themes were identified and organized into nine main categories: the context in which the request for transition arises, the wording used in the disclosure, the onset of the transgender identity request, difficulties associated with puberty, the young person’s sexual orientation, the influence of digital and real social networks, the transidentity consultation process, parental reactions, and finally, the psychological state of the young person post-disclosure. Each of these themes will be explored in detail to better understand the complexity of the experiences shared by the parents.

Where possible, the presentation of these themes includes a descriptive statistical analysis of our sample, particularly in terms of the prevalence and proportion of AYAs affected by specific traits. These data must be interpreted with caution, given the heterogeneity of the statements and the subjective nature of parental accounts.

Furthermore, the data from our sample should not be generalized to the entire population affected by these issues. Instead, these findings aim to provide insight into the subjective experiences of parents navigating their adolescent’s disclosure of transgender identity, offering a nuanced perspective on the diverse challenges and emotions involved.

#### Background to the Transition Request

The family context reveals several significant aspects (see Table 2). A notable finding is the prevalence of long-term psychological care (63% of our sample), indicating a history of mental health issues or psychological vulnerability in the adolescent or young adult and, in some cases, within the family. Additionally, a comorbid psychiatric diagnosis was reported in 37% of cases, with 8% of these diagnoses made during hospitalization. The most frequently reported conditions include depression (20%), autism spectrum disorders (17%), eating disorders (13%), school phobia (10%), and, less frequently, schizophrenia (3%) and post-traumatic stress disorder (2%). These findings are consistent with the literature on comorbidities commonly identified in young people experiencing gender dysphoria.

**Table 2:** Psychopathology, comorbidities, and contributing factors (*n* = 60)

Category	Subcategory (*n*)	%	Total % per category
Preliminary monitoring (38)	Prior psychological counseling (38)	63	63%
	Prior hospitalizations (5)	8	
Psychiatric diagnosis (18)	Depression (12)	20	30%
	Autism Spectrum Disorder (10)	17	
	Eating Disorder (8)	13	
	School phobia (6)	10	
	Schizophrenia (2)	3	
	Post-Traumatic Stress Disorder (1)	2	
Comorbidities and/or contributing factors (43)	Separations (16)	27	72%
	Self-cutting (13)	22	
	Suicidality (13)	22	
	High intellectual potential (9)	15	
	Rape (8)	13	
	Harassment (7)	12	
	Domestic violence (6)	10	
	Bereavement (5)	8	
	Addiction (4)	7	
	Serious accidents (3)	5	

Furthermore, 72% of parents reported concerns related to mental health difficulties, including suicidality (22% of those with comorbidities), self-harm (22%), and substance use disorders (7%). Somatic comorbidities related to physical health conditions, surgeries, or accidents were also mentioned (5%).

Many parents highlighted significant life events (reported in 60% of cases) that coincided with or preceded their child’s disclosure of transgender identity. These events include parental separation or divorce (27%), experiences of sexual assault (13%), bullying at school (12%), various forms of domestic violence (10%), and bereavement (8%). While not classified as comorbidities, these life stressors may contribute to the broader psychological context in which identity-related questions emerge.

Interestingly, 15% of parents reported that their child had been identified as having high intellectual potential following psychological assessments. Parents often described their children as introspective, academically gifted, and socially reserved prior to adolescence: “We had her evaluated by a psychologist specializing in gifted children around the age of eight. While I’m unsure of the thoroughness of the assessment, the psychologist confirmed her high potential.”

These findings align with previous studies indicating that a significant proportion of adolescents seeking gender-related care have a history of mental health concerns unrelated to gender dysphoria. For example, Kaltiala-Heino et al. (2015) reported that 75% of applicants had received or were receiving psychiatric treatment for issues other than gender dysphoria. Other studies have documented mental health comorbidities in 71–74.5% of transgender youth (Becerra-Culqui et al., 2018; Bechard et al., 2017; D’Angelo et al., 2021; de Vries et al., 2010; Hall et al., 2021; Kozlowska et al., 2021).

While these life events and psychological vulnerabilities are not unique to transgender-identifying youth, they may shape the ways in which some individuals interpret their distress and seek meaning in their experiences. Identity formation in adolescence is a dynamic process influenced by both personal history and external context. For some young people, adopting a transgender identity may provide a framework through which they attempt to make sense of their emotional struggles, particularly when other explanations feel inadequate or unavailable. This perspective does not negate the reality of gender dysphoria but highlights the importance of considering a broad range of psychological and social factors when supporting youth through their identity exploration.

#### Wording of the Disclosure

Parents expressed a deep sense of incomprehension and powerlessness following their child’s disclosure of transgender identity. They reported uncertainty about how to react, as the disclosure was often accompanied by requests from their child for changes in name and pronouns, which implied significant shifts in family dynamics and relationships.

Many parents described feeling conflicted, as these requests challenged their understanding of their child’s identity. Some parents perceived the disclosure as lacking spontaneity or authenticity, noting that their child’s behavior seemed rehearsed or influenced by external sources. They described difficulties in maintaining open dialogue, as conversations often became tense, fragmented, or entirely avoided following the disclosure.

The accounts revealed that disclosures were frequently made in writing—via letters, emails, or text messages—rather than through face-to-face conversations. Parents observed that the language used in these written communications often mirrored terminology and expressions commonly found on online forums or social media platforms focused on gender identity. Some parents recognized similarities between their child’s message and publicly available templates or guidance materials found online: “One day, [X] sent us a very detailed email with extensive terminology about being transgender and seeking medical transition. [X] even provided links to specific resources”.

Following the disclosure, many parents reported that it became challenging to engage in discussions about their child’s identity. Attempts to ask questions or express concerns were sometimes met with accusations of misunderstanding or intolerance. Parents described a communication dynamic where expressions of doubt were perceived as invalidating, creating barriers to open dialogue. Some parents interpreted this as a defense mechanism, reflecting their child’s need to assert a clear narrative amidst internal uncertainty.

In these accounts, parents noted discrepancies between their child’s pre-disclosure behavior and the certainty expressed post-disclosure. They observed that their child’s explanations often lacked depth or appeared inconsistent, leading them to believe that identity-related discussions served to manage broader emotional distress. In some cases, the term “transphobia” was described as being used in family conflicts to dismiss parental concerns, which parents perceived as an obstacle to constructive communication.

#### Triggers for the Transition Request

Parents identified several potential triggers for the onset of the transition request. They cited puberty-related difficulties and the profound impact of the COVID-19 lockdown, which intensified feelings of isolation and increased engagement with social networks: “The lockdown begins, and X spends all her time on social networks.” Other noted triggers include relationship issues with peers, romantic disappointments, and the growing influence of friends and digital platforms.

Parents emphasized these potential triggers in their written statements to highlight the importance of considering the broader context in which the disclosure occurred. They described their children as vulnerable individuals navigating adolescence, often facing emotional challenges, feelings of isolation, and shifts in self-image. This contextualization helped parents make sense of what they perceived as the sudden and unexpected nature of their child’s request.

Notably, many parents described their child’s transition request as characterized by three recurring features: its unexpected nature (e.g., “no warning signs,” “no precursor signs,” “no possible explanation,” “incomprehensible”), its sudden onset (e.g., “overnight”), and the urgency expressed in the desire for change (e.g., “quickly,” “urgently,” “immediately”). Parents often reported experiencing feelings of surprise, shock, and astonishment in response to these disclosures, which they associated with the rapid progression from self-identification to requests for social or medical transition.

#### Puberty-Related Difficulties

Puberty, a period of significant biological and psychological transition, is often marked by identity exploration and self-discovery. During this challenging developmental phase, many parents in our sample reported that their child disclosed a transgender identity, often described as sudden and unexpected. This stage is characterized by emotional fluctuations, identity questioning, and a search for self-acceptance, which, while complex, aligns with typical adolescent developmental processes.

Parents frequently highlighted the impact of bodily changes during puberty, which sometimes led to issues such as body dysmorphic concerns, eating disorders like anorexia—primarily observed in natal females identifying as transmale in our sample—and difficulties coping with early or accelerated pubertal development. For example, one parent noted: “After recovering from anorexia, she also went through a period of letting go, involving excessive consumption of food, as well as alcohol, and experiences with older boys.”

For many adolescents, particularly girls, the emergence of body-related anxieties seemed to be triggered by the psychological adjustments associated with puberty. These concerns were often intensified by external stressors, such as relocating, experiences of bullying, or relationship breakups. Some parents described how their children’s negative self-perception evolved during this period, with identity-related struggles emerging alongside broader emotional distress.

In several accounts, parents observed that their child’s exploration of transgender identity provided a framework for understanding and managing these anxieties. This identification was described as offering a sense of coherence or relief in the face of emotional discomfort. In this way, the discourse surrounding gender identity was perceived by some parents as a coping mechanism—a means of articulating and addressing feelings of distress during a turbulent developmental stage.

#### Young Person’s Sexual Orientation

The issue of young people’s sexual orientation is central to many of the parents’ accounts. A recurring pattern emerged: several young people who later identified as transgender had initially come out as homosexual, an announcement generally welcomed with acceptance by the parents in our sample. This trajectory of identity exploration often appeared to follow a progression, starting with a declaration of homosexuality and later evolving toward identifications such as non-binary, bisexual, pansexual, or asexual.

Additionally, many parents noted that their children generally did not have an active sexual life before expressing a desire to transition. For example, one parent shared: “She is a young girl who has never had any issues with her body until now: puberty started in early sixth grade and was well accepted; she was very athletic and dressed quite attractively throughout middle school. However, she has never had relationships with boys. It is quite possible that she is actually homosexual.”

These observations reflect parents’ attempts to understand their child’s evolving identity within the broader context of adolescent development. They often described their children’s identity exploration as part of a dynamic process, influenced by personal experiences, social interactions, and developmental factors.

#### The Influence of Digital and Real Social Networks

The influence of social networks, both digital and real, was frequently discussed by parents, who viewed these platforms as having a significant role in shaping their child’s understanding of gender identity. Some parents perceived these networks as spaces where gender identity exploration was facilitated, while others expressed concerns about the potential for ideological reinforcement. It is important to note that these perceptions reflect the subjective experiences and interpretations of the parents in our sample.

In many cases, parents suggested that their child’s identification as transgender may have been influenced by interactions with online communities or peers sharing gender-affirming narratives. This influence was particularly noted when young people had close relationships with friends undergoing similar transitions or were part of school environments with visible transgender communities. Parents often described these environments as providing a framework that helped their child articulate and express feelings related to their identity. In certain contexts, healthcare professionals also contributed by framing the child’s distress within the lens of gender dysphoria, which parents felt could shape their child’s understanding of their experiences.

More specifically, parents frequently mentioned the role of online forums dedicated to transgender topics, websites offering self-assessment tools, and the pervasive influence of social media platforms such as Instagram, TikTok, and YouTube. For example, one parent shared: “We then realized that, through the internet, he was in contact with an American ‘trans’ forum that he considers his ‘community.’ He has adopted all the language elements of this new vocabulary.”

These platforms often featured activists, influencers, and peer communities, which some parents perceived as contributing to the adoption of identity-related terminology and concepts. Another parent noted: “Notably on TikTok, I was surprised by what I found there… I am neither homophobic nor transphobic, and I consider myself to be quite open-minded, but I felt that this platform had been strongly influenced by LGBTQIA+ content.”

While these accounts reflect the perspectives of the parents in our study, it is important to recognize that peer and online communities also provide critical spaces for support, validation, and identity exploration—common processes during adolescence. The diversity of these experiences underscores the complex ways in which young people navigate identity development within contemporary social and digital landscapes.

#### Transidentity Consultation Procedure

According to the accounts, the experience of specialized transidentity consultations, which occurred in 20 out of 60 cases (approximately one-third of the families, see Table 3), raised several concerns and criticisms. Parents often felt that these consultations were systematically biased toward affirming transgender identity. Additionally, many expressed frustration and concern about being excluded from discussions regarding their child’s care. Specifically, 55% of parents (11 out of 20 families) reported not being included in the consultations at all, while 40% (8 out of 20 families) were included only once.

**Table 3:** Parental experiences of specialist consultations (*n* = 20)

Experiences	%
Specialist transidentity consultation (20/60)	33.3
Parents not admitted for discussions (11/20)	55
Parents seen only once (8/20)	40
Diagnosis made after 1 or 2 appointments (15/20)	75
Appointments of less than 15 min (5/20)	25
Psychological pressure (3/20)	15
Transition details given at first appointment (9/20)	45

Several parents also felt that their child’s overall psychological context and prior experiences were not sufficiently considered during these consultations. A major concern was the speed of the diagnostic process: 75% of parents (15 out of 20 families) reported that one or two appointments were deemed sufficient to establish a gender dysphoria diagnosis and authorize medical interventions. Moreover, 25% (5 out of 20 families) described these appointments as unusually brief, lasting less than 15 min each.

Some parents highlighted practices they perceived as inappropriate. For example, 15% of families (3 out of 20 cases) reported experiencing psychological pressure, including references to potential suicide risks, which they felt were used to influence parental decisions regarding support for transition. Additionally, 45% (9 out of 20 families) noted that initial consultations often focused primarily on the practical steps of transitioning, with little exploration of the underlying factors contributing to the child’s distress.

Parents expressed difficulties in finding mental health professionals who prioritized understanding the reasons behind their child’s desire to transition, rather than automatically supporting the transition process. One parent shared: “I find it extremely difficult to find psychiatrists who are primarily concerned with listening and exploring the reasons for this desire for transition, rather than providing systematic support for transition.”

In some cases, parents recounted situations where professionals appeared to encourage distancing from family members, which they found troubling. For instance, one parent reported that a psychiatrist advised their child to cut ties with what was described as an ‘abusive’ family environment, which the parent felt was an unjust characterization. Another parent expressed distress after encountering a professional who discouraged any inquiry into potential causes or contributing factors related to the child’s transgender identity: “She told me not to look for causes or antecedents and ended with this sentence: ‘Ask yourself why you are resisting instead’.”

These accounts reflect a range of parental concerns regarding the clinical approaches encountered in specialized consultations, particularly related to the perceived lack of comprehensive psychological assessment and limited family involvement in the decision-making process.

#### Parental Reaction

Faced with their child’s desire to transition, parents often experience significant ambivalence. They find themselves torn between the wish to support their child, maintaining open dialogue and a strong relationship, and the fear of potential consequences associated with medical interventions. One parent reflected this tension: “How can I open the dialogue with my daughter so that she takes time to reflect more before compromising herself with heavy and difficult medical procedures?”

Parents frequently express anxiety about their child’s well-being, particularly concerning the possibility of regret after undergoing medical transition, and the long-term impacts on their child’s health, personal development, and future life choices. A common source of distress is the fear of not being able to provide adequate protection when their child is perceived as too young or vulnerable to make such life-altering decisions.

Another recurrent theme is the feeling of isolation parents experience when seeking support from healthcare professionals. Some parents reported that medical consultations often focused on affirming the child’s expressed identity without addressing parental concerns: “We could see a psychiatrist very quickly who clearly recommended supporting our daughter in her gender transition because it was her choice; however, we did not receive the listening or support we expected.”

This sense of loneliness is compounded by the perceived lack of space for parental reflection and discussion within the healthcare system. Parents often feel that their concerns are misunderstood or dismissed, leaving them to navigate complex emotions and uncertainties about how best to support their child while safeguarding their long-term well-being.

#### Young People’s Psychological State After the Announcement

Parents frequently reported that their child’s psychological well-being deteriorated after disclosing their desire to transition. In all cases (100%), parents noted signs of increased distress, which manifested as heightened psychological symptoms, changes in behavior, strained family relationships, and reduced engagement in school activities (see Table 4).

**Table 4:** Description of post-passing behavioral deterioration observed by parents

Category (*n*)	%
School dropouts (10)	16.7
Withdrawal (9)	15
Social anxiety (8)	13.3
Refusal to talk (6)	10
Increased aggression (3)	5
Self-cutting (4)	6.7

Young people often distanced themselves from their families following the disclosure, preferring written communication over direct dialogue. Parents observed that their children adopted discourse patterns that seemed pre-structured, making it challenging to engage in open discussions about their feelings and motivations. One parent shared: “Our son became vehemently opposed to any discussion, labeling us as ‘transphobic’ through a radical discourse, likely influenced by content encountered on social media.”

Some parents expressed feelings of helplessness after agreeing to support their child’s transition, only to witness further declines in their child’s well-being. Reports included reduced academic performance, withdrawal from previously enjoyed activities, and breakdowns in family relationships. Communication often became tense, with conflicts escalating through digital platforms such as text messages or emails.

The data highlighted concerning trends, including increased school dropout rates, symptoms of abulia, severe depression, and a pervasive sense of hopelessness. Other alarming signs reported by parents included heightened dependence on social media, mood instability, suicidal ideation or attempts, and, in some cases, accusations of parental misconduct. A few accounts described situations where young people used threats of estrangement to pressure parents into consenting to medical interventions.

Parents described profound emotional distress and breakdowns in communication with their children. As one parent painfully recalled: “I’ve never felt as distant from her as I did when she came out as a trans man.” Another parent shared: “Cold, distant, aggressive, very violent with me. He says, ‘Your physical presence by my side is hell,’ and has suicidal urges.”

These accounts underscore the complexity of family dynamics following the disclosure of transgender identity, highlighting the need for supportive interventions that address both the young person’s and the family’s psychological well-being.

#### Incidental Results

Unexpected findings from this study revealed that some young people who had initially expressed a desire to transition eventually chose to discontinue this process. Notably, two individuals, both diagnosed with severe conditions—autism in one case and schizophrenia in the other—reported a sense of relief after deciding not to pursue transition. One of them described feeling liberated from what was referred to as a “misguided path.” Additionally, one participant acknowledged having constructed their narrative around transidentity based on information encountered online, using it as a framework to make sense of personal distress.

These incidental results highlight the complexity of identity exploration and underscore the importance of comprehensive psychological support that considers the diverse trajectories young people may experience during this process.

### Summary

The findings of this study highlight the diverse and complex experiences of parents navigating their child’s disclosure of transgender identity. Common themes include parental ambivalence, concerns about the rapidity of medical interventions, feelings of exclusion from clinical decision-making, and the emotional strain within family relationships. The deterioration of the young person’s psychological well-being after disclosure, as reported by parents, raises questions about the adequacy of current support systems. Additionally, incidental cases of desistance emphasize the fluidity of identity exploration in adolescence. These results underscore the need for holistic, individualized approaches that consider both the young person’s and the family’s psychological well-being, providing a foundation for deeper reflection in the discussion section.

## Discussion

The primary aim of this study was to thoroughly explore the experiences and perceptions of parents of AYA who identify as transgender. Specifically, this study aimed to understand the underlying reasons for the hesitation some parents show in supporting their child’s transgender identity, despite their expressed desire to be supportive. Through an analysis of the collected testimonies, we identified key themes that reveal the complexity of this experience, highlighting emotional, psychological, and social challenges faced by both parents and AYAs throughout the transition process. Our philosophical perspective on the subject of trans identified adolescents is grounded in a clinical approach attentive to psychic development, identity construction, and the need for careful, evidence-based evaluation in the face of contemporary ideological pressures.

### Perception of Inconsistency and Ideological Influence in the Child’s Statement

Many parents expressed a sense of discrepancy between their child’s disclosure of transidentity and their prior understanding of their child. They often perceived these statements as reflecting pre-formatted discourse, potentially influenced by online sources, without prior indicators of gender-related distress in the child’s history. This sudden shift in narrative appeared to some parents as a break in the continuity of their child’s identity development. Additionally, parents reported feeling that their children’s views were shaped by external discourses characterized by specific terminology and digital social interactions. These influences were perceived as barriers to meaningful dialogue, with conversations becoming repetitive and difficult to navigate (Littman, 2018).

### Parents Feeling Left Behind or Unsupported

Parents frequently experienced feelings of isolation and powerlessness regarding their situation (Hefez, 2016). They reported challenges in maintaining open communication with their child (Chiland, 2014), exacerbated by social taboos and difficulties accessing neutral, supportive professional guidance. Many parents described specialized consultations as focusing primarily on facilitating the transition process, often without a comprehensive psychological assessment. Some parents raised concerns about rapid diagnostic conclusions, such as cases where gender dysphoria was diagnosed without sufficient consideration of coexisting conditions like autism. These experiences raise important questions about the adequacy of current clinical practices in addressing the multifaceted needs of young people (Malpas & Bosman, 2014).

These experiences of isolation often intersect with deeper concerns about the psychological underpinnings of their child’s identification, as explored in the next section.

### The Perception of Transidentification as a Symptom

Parents frequently perceived their child’s transidentification as a possible expression of underlying psychological distress, encompassing challenges related to body image, identity, sexuality, or broader emotional difficulties. In this context, transidentification was not solely viewed as an act of self-affirmation but also as a coping mechanism to manage deeper emotional struggles. This included responses to feelings of bodily discomfort, trauma, or difficulties navigating adolescence and peer relationships (Diaz & Bailey, 2025).

One notable observation was the approach adopted in specialized transidentity consultations. Testimonies indicated that initial consultations often prioritized discussions about social and medical transition steps over comprehensive psychological assessments. Additionally, many parents expressed concerns about the limited involvement of child psychiatry specialists, reflecting broader challenges within mental health services for youth in France (Cour des Comptes, 2023).

### Study Limitations

Our study acknowledges several limitations that should be considered when interpreting the results. First, while our sample provides valuable insights, its size and composition limit the generalizability of the findings. The participants were self-selected, comprising parents who voluntarily shared their concerns regarding their child’s disclosure of transgender identity. This introduces a potential selection bias, as these experiences may not represent the full spectrum of parental responses, particularly those of families who have had neutral or positive experiences with their child’s transition.

Furthermore, the method of collecting testimonies via email may have influenced the content and tone of the narratives. Written accounts can vary in depth, detail, and emotional expression compared to in-person interviews, potentially limiting the richness of the data. Additionally, the lack of direct interaction with researchers may have restricted opportunities for clarification or elaboration on key points.

Although our qualitative analysis was conducted rigorously, it remains inherently subjective, influenced by the researchers’ interpretations and potential biases in identifying and analyzing themes. To mitigate this, multiple researchers were involved in the coding process, and discrepancies were discussed and resolved collectively. Nonetheless, the subjective nature of both the testimonies and the analysis should be considered when interpreting the findings.

Finally, the homogeneous nature of the sample, particularly regarding the predominance of mothers and the specific demographic characteristics of the families, may limit the applicability of the results to more diverse populations. Future research should aim to include a broader range of family structures, cultural backgrounds, and parental perspectives to provide a more comprehensive understanding of the experiences of families navigating issues related to transidentity.

### Conclusion

In summary, the findings highlight the multifaceted challenges parents face in navigating their child’s transgender identification, shaped by emotional, psychological, and systemic factors. Parents reported feelings of ambivalence, isolation, and concerns about the adequacy of clinical support, particularly regarding comprehensive psychological assessments. These insights underscore the need for more nuanced, supportive, and individualized clinical approaches that address both the young person’s and the family’s well-being. The study also calls for further research to explore diverse family experiences and to develop best practices for supporting both transgender youth and their families within a holistic framework.

### Recommendations

#### Transidentification: Understanding Parental Concerns for Better Psychological Support

The exploration of parental testimonies reveals the complex emotional landscape experienced by parents confronting their child’s disclosure of transidentity. This emotional response, far from being rooted in simple opposition, often emerges from a genuine desire to support their child while navigating profound uncertainties. Our study identifies five critical areas of concern that merit attention to improve psychological support for both parents and young people.

1. Parental Response to Transidentity
  Transidentity, like many identity-related issues, significantly impacts family dynamics. Parents frequently find themselves balancing the desire to provide unwavering support with concerns about the potential long-term implications of medical transitions. The disclosure of transidentity can create relational tensions, underscoring the importance of tailored psychological support to foster constructive dialogue within families and strengthen parent–child relationships.
2. Parental Availability and Engagement
  Despite the emotional challenges posed by their child’s disclosure, many parents adopt a proactive stance. They actively seek information, resources, and professional guidance to better understand their child’s experience. This engagement reflects not resistance but a willingness to support their child thoughtfully. It highlights the need for access to reliable, balanced, and evidence-based information to guide families through these complex situations.
3. The Importance of Dialogue
  Open and empathetic communication is crucial for fostering understanding between parents and their children. Adolescents may view their transidentity as a response to emotional distress, while parents, striving to make sense of this, can inadvertently create communication barriers. Clinicians play a key role in facilitating these conversations, promoting environments where both young people and their parents feel heard and supported. However, many parents in our study reported feeling sidelined in specialized consultations, pointing to the need for more inclusive clinical practices.
4. Parents’ Concerns About Psychological Well-Being
  Parents often express concerns about the psychological well-being of their children post-disclosure, particularly when transidentity intersects with pre-existing mental health conditions. These concerns are not inherently oppositional but reflect a deep care for their child’s long-term health and happiness. Clinical encounters that dismiss these concerns can exacerbate parental anxiety. A more nuanced clinical approach, grounded in comprehensive psychological assessment, is essential to address both the adolescent’s and the family’s needs (Kaltiala et al., 2023).
5. Social Impact and Comorbidities
  Some testimonies highlight the role of external influences, such as social media or peer dynamics, in shaping young people’s identities. This raises important questions about the interplay between environmental factors and personal identity development. While these factors do not diminish the validity of transidentification, they underscore the need for careful, individualized assessments that consider the broader psychosocial context.

#### Recommendations for Comprehensive Support

In conclusion, a comprehensive, psychotherapeutic approach should be the cornerstone of initial support for families navigating transidentity. Psychological support can help families process their emotions, foster open communication, and ensure that decisions regarding transition are made thoughtfully and collaboratively. This approach does not preclude medical interventions but situates them within a broader framework of psychosocial care.

Confrontational or dismissive attitudes may hinder the adolescent’s sense of security, while a compassionate, gradual approach that acknowledges both the young person’s experiences and the parents’ concerns fosters healthier family dynamics. The challenges posed by transidentity require an interdisciplinary approach that integrates medical, psychological, and social perspectives to ensure holistic care.

Our findings emphasize the urgent need for psychotherapeutic strategies that prioritize exploration and understanding over immediate affirmation. This model supports the adolescent’s developmental journey while safeguarding against premature decisions that may overlook underlying psychological needs.

These insights align with the principles outlined in the Cass Review (Cass, 2024), which advocates for comprehensive psychosocial assessments before initiating medical interventions. By adopting this holistic approach, we can better support families in navigating the complexities of transidentity, promoting the well-being of both young people and their parents.

## Footnotes

1 https://www.observatoirepetitesirene.org/. The OPS is an association created in 2021 by a multi-disciplinary group of professionals: medical doctors, psychologists, lawyers, psychoanalysts, and teachers, with the aim to warn childcare professionals about the impact of social networks and militancy on children’s development.
